# Modularized Reconfigurable Functional Electromagnetic Surfaces Using Tightly Coupled Antennas and Back-Loaded Radio Frequency Circuits

**DOI:** 10.3390/mi15121490

**Published:** 2024-12-12

**Authors:** Boyu Sima, Jiayi Gong, Zhenghu Xi, Shunli Zhang, Ziling Li, Tao Wang, Guoxiao Cheng, Huangyan Li, Xiang Wang, Jianpeng Wang, Zhiyuan Zong

**Affiliations:** 1 Key Laboratory of Near-Range RF Sensing ICs and Microsystems (NJUST), Ministry of Education, School of Electronic and Optical Engineering, Nanjing University of Science and Technology, Nanjing 210094, China; smby@njust.edu.cn (B.S.); gongjiayi@njust.edu.cn (J.G.);; 2 Shanghai Radio Equipment Research Institute, Shanghai Academy of Spaceflight Technology, Shanghai 201109, China

**Keywords:** electromagnetic surfaces, tightly coupled dipole antenna, broadband absorber, broadband polarization conversion, reconfigurable, modularized

## Abstract

This paper presents a modularized reconfigurable functional electromagnetic surface (MRFES) for broadband absorption and polarization conversion by using tightly coupled dipole antennas (TCDA) and back-loaded radio frequency (RF) circuits (BLRFC). A dual-polarized antenna array with tight coupling and wide angular scanning characteristics is designed. By loading different RF circuits on the back side of the antenna array’s ground plane, switchable broadband absorption and polarization conversion functions are achieved. The design adopts modularization to facilitate the replacement of back-loaded RF circuits for diverse electromagnetic (EM) control functions. The final design of the tightly coupled antenna array has a thickness of 13.437 mm and a size of 119.5 mm × 119.5 mm. It works in a bandwidth range of 4.14–13 GHz. Upon loading the absorption circuit board, a broadband absorbing electromagnetic (EM) surface is formed, achieving dual-polarization absorption within a bandwidth of 4.14–12.4 GHz. With the polarization conversion circuit board attached, polarization conversion effects are realized within a bandwidth of 4.4–12.9 GHz. Both simulations and experiments verify that the designed EM surface possesses modular reconfigurable functions for broadband absorption/polarization conversion. The proposed design scheme holds promising prospects for applications in active stealth, adaptive camouflage, intelligent communication and other fields.

## 1. Introduction

Electromagnetic (EM) surfaces, constituted by subwavelength elements arranged in periodic or quasi-periodic two-dimensional arrays, have garnered substantial interest in the electromagnetics community in recent years. Among these, metasurfaces [1,2] and frequency selective surfaces (FSSs) [3,4] represent two critical research directions. By ingeniously designing the subwavelength structures, it is possible to achieve multidimensional and high-degree-of-freedom control over incident EM waves, encompassing functions such as absorption, filtering, anomalous reflection, anomalous transmission, the polarization conversion, and beam steering [5,6,7,8,9,10,11]. Owing to their unique and multi-functional characteristics, these structures are broadly applicable in communications, sensing, and electronic countermeasures. For example, Li et al. proposed a multi-functional metasurface (MFMS), based on graphene and photosensitive silicon (Si), which integrates three functions: broadband absorption, broadband linear and circular polarization conversions in the THz band [12]. Mutlu et al. designed bi-functional metasurfaces comprising U-shaped subwavelength resonators and a middle grid (rectangular-hole array), potentially enabling both symmetric and asymmetric PPR functionalities in one microwave structure at the subwavelength scale [13].

Reconfigurable EM surfaces that can change the manipulation function of incident waves have experienced rapid development in recent years [14,15], demonstrating significant potential in wireless communications, radar stealth, and photonic devices [16,17]. To meet the requirement of different application scenarios, various reconfigurable EM surfaces have been developed, such as beam scanning reflect array, polarization reconfigurable metasurface, frequency tunable FSS, etc. [18,19,20,21,22]. Various methodologies have been explored to realize reconfigurable electromagnetic response properties, including the use of diodes, micro-electromechanical system (MEMS) switches and diverse radio frequency (RF) switches [23,24,25,26,27,28]. A pivotal research direction in reconfigurable functional EM metasurfaces is the realization of broadband reconfigurability [29]. This is of significant value for effective spectrum management and utilization, albeit challenging to achieve.

In this paper, a design of modularized reconfigurable functional electromagnetic surfaces (MRFESs) for broadband absorption and polarization conversion by using tightly coupled dipole antennas (TCDA) and back-loaded radio frequency circuits (BLRFC) is proposed. Compared with traditional BLRFC-based metasurfaces [30,31,32,33], the design of the proposed MRFES is reconfigurable. Its modular design can achieve mechanical switching, making it easier to achieve broadband effects and reducing the need for high-performance chips and DC bias circuits. Initially, a new TCDA array is designed and implemented, one which has the characteristics of simple structure, independence, low profile and being ultra-wideband. On the basis of this antenna array, by loading different functional RF circuits on the back side of the ground plane, switchable EM wave manipulation functions with broadband characteristics are achieved. The scheme employs a modular design, enabling the realization of diverse EM modulation functions through the convenient replacement of the rear mounted RF circuits. For typical verification, two EM wave modulation states are realized by conveniently replacing the functional back-loaded RF circuits in this work. In the absorption state, the incident wave is received by the front broadband antenna array and transmitted to the absorbing circuit board connected to the back side of the ground of the antenna array. The absorbing circuit board is adjusted to better match the working bandwidth of the antenna, thereby obtaining a broadband absorbing effect. In the polarization conversion state, an inter connection circuit board is connected to the back of the TCDA array, replacing the absorbing circuit board to achieve the broadband polarization conversion effect. The connection between the tightly coupled antenna array board and the back-connected RF circuit board adopts smooth-bore SMP adapter components, which can be easily disassembled. Owing to its advantages of ultra-wide bandwidth, modularity, reconfigurability, simplified structure and high performance, the design of the proposed MRFES has a wide application prospect in military and civil fields, such as electromagnetic compatibility and electromagnetic shielding, and also as a polarization converter, radome, absorber, corner reflector, etc.

## 2. Design and Simulation

### 2.1. Design Principle

**Figure 1:**
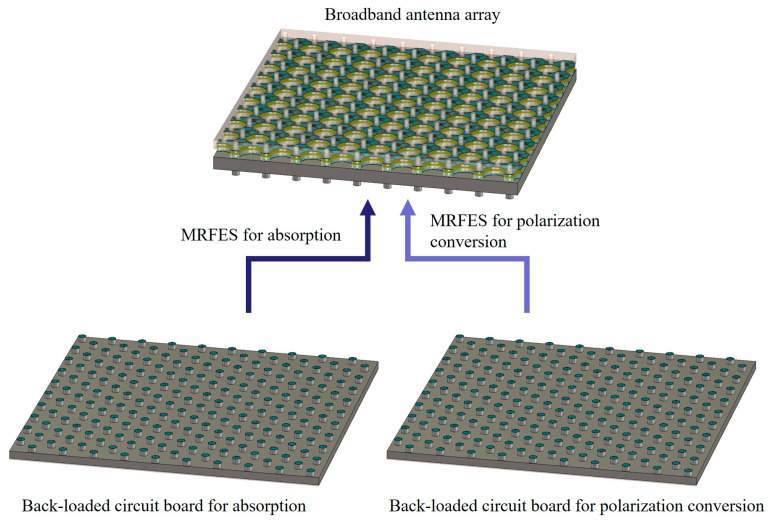
Three-dimensional schematics diagram of the proposed MRFES.

**Figure 2:**
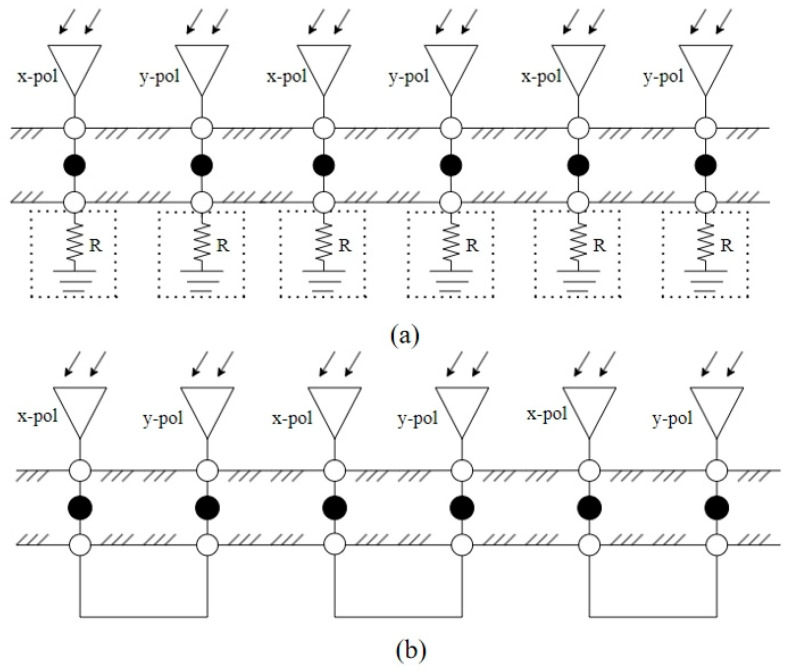
Equivalent structural diagram of the proposed MRFES for (**a**) absorption and (**b**) polarization conversion.

In this paper, a design for an MRFES with broadband characteristics based on a TCDA and back-loaded RF circuits is proposed. The three-dimensional schematics diagram structure is illustrated in Figure 1, consisting of a tightly coupled wideband antenna array and back-connected RF circuit boards which can be switched like a module to realize different EM wave manipulation functions. The TCDA is designed to receive incident EM waves in an ultra-wide frequency band and couple them into coaxial feeders converting spatial EM waves into guided waves. This is one of the key points as to why this design can achieve broadband characteristics. To minimize the thickness of the structure, we specially design and implement a new TCDA, one which can provide a wide enough bandwidth and a good impedance matching effect with a relatively low profile. On the basis of this TCDA, a wideband reconfigurable functional EM surface can be designed and realized by connecting RF circuits with different functions on the back side of the antenna’s ground. A broadband absorbing circuit board and a polarization conversion circuit board is adapted to the TCDA in order to realize switchable broadband absorption and polarization conversion capacity. The TCDA antenna array and the circuit board are connected through an optical hole SMP connector. With the utilization of smooth-bore SMP connectors, the process of mechanically switching the circuit board is easy to disassemble, and it is more convenient to replace the back-connected RF circuit to realize different EM wave control functions. The schematic diagram of the principle structure of the absorbing EM metasurface and the polarization conversion EM surface constructed in this way is shown in Figure 2a,b. When the antenna array is connected to the absorbing circuit board, the whole MRFES is served as an absorptive surface. The input EM waves received by the TCDA antenna array for both X and Y polarizations are absorbed by the resistors loaded at the terminal of the absorbing circuit. When the antenna array is connected to the polarization conversion circuit board, the whole MRFES is served as a polarization conversion surface. The input EM waves received by the TCDA antenna array for X polarization are transmitted to the port for Y polarization and vice versa to realize the polarization conversion effect. The switching between broadband absorbing and polarization conversion functions realize the replacement of the circuit board with the corresponding EM wave manipulation effect. In this design scheme, we adopt the reconfigurable method of mechanical connection in order to realize the switching of a different circuit board, offering the advantages of simple structure, independence, low profile, ultra-wide band, modular design, etc. This is applicable in scenarios where the switching of EM wave control functions is necessary, but where real-time and rapid reconfiguration are not imperative.

### 2.2. Design and Simulation of TCDA Unit Structure

**Figure 3:**
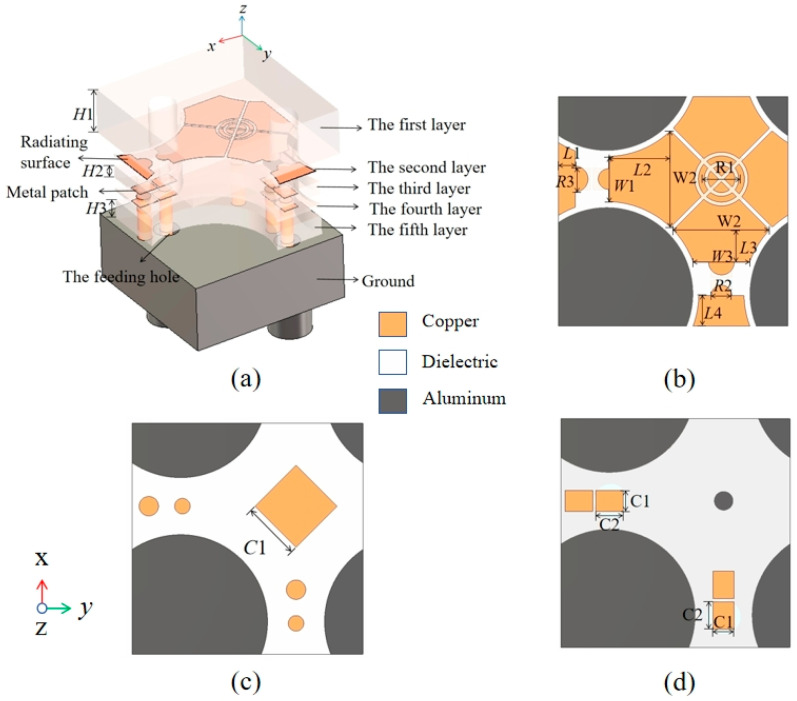
(**a**) The 3D diagram of the TCDA unit structure, (**b**) the top view of the second layer, (**c**) the third layer, and (**d**) the fourth layer.

**Figure 4:**
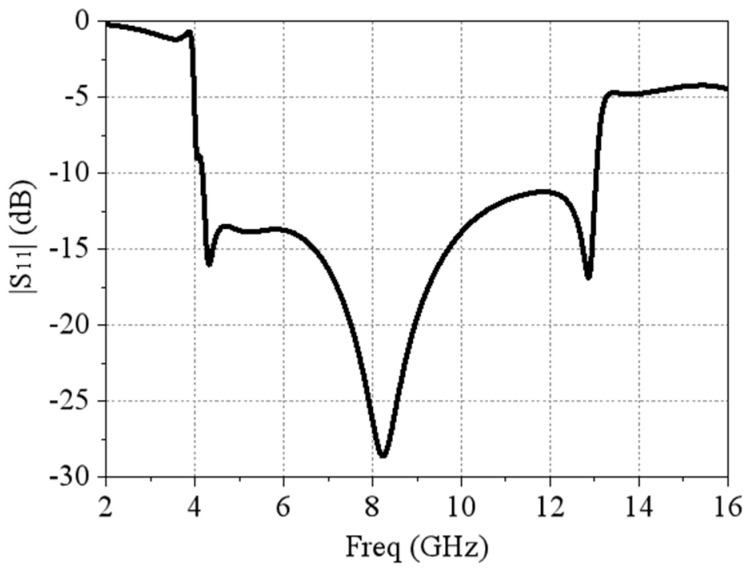
The simulated |$S_{11}$| results of the TCDA unit.

As mentioned earlier, the proposed MRFES is mainly composed of two parts, namely the dual-polarized broadband TCDA and the back-connected RF circuit board. The size of our proposed TCDA element is designed to ensure a low profile and a wide band. Therefore, the antenna length and width of the proposed TCDA unit structure are each 11.95 mm and the height is 7.01 mm. The unit cell is 0.165$\lambda_{L}$ × 0.165$\lambda_{L}$ × 0.095$\lambda_{L}$ at the lowest frequency of operation ($\lambda_{L}$ is the wavelength of the lowest frequency in space). As shown in Figure 3a, the TCDA unit structure is composed of a multi-layer dielectric. The uppermost layer is the first layer and the F4B plate is made of F4B, with a dielectric constant *ε*r = 3.55, which serves the function of adjusting impedance matching and expanding the scanning angle range. The rest of the antenna dielectric layers are made of FR4 plates, with a dielectric constant of 2.2. In order to achieve a wider operating frequency band, we adopt the unbalanced feeding method. As shown in Figure 3b, the radiating layer of the unbalanced feeding structure is composed of dual-polarized dipoles and the main radiating surface of the broadband antenna is a dual-polarized dipole array. The design is printed on the upper surface of the second dielectric plate. At the intersection of horizontal and vertical dipoles on the lower surface of the second dielectric plate, a rectangular metal patch is printed, as shown in Figure 3c. Due to the use of the unbalanced feeding mode, the currents of two feeders conducted by metal columns will have opposite directions and different sizes. When the surface of the dipole antenna is excited, a certain amount of common mode resonance will be produced. We add short-circuit holes and a rectangular metal-to-ground radiating surface to suppress and transfer the common mode resonance. A metal via is arranged at the position of the second dielectric plate and is connected to the ground under the array in order to eliminate the common mode resonance and broaden the high frequency. The upper surface of the third dielectric plate layer is similarly printed with a rectangular metal patch at the corresponding position of the second layer, and the short-circuit hole is also used to connect the ground plate and the dipole sub-layer to form electrical coupling with the second layer rectangular metal in order to strengthen the common mode resonance removal. The lower surface is also printed with metal patches, located respectively at either side of the feeding hole and the short-circuit hole, while the metal patch has gaps at either side of the feeding hole and the short-circuit hole. The metal patch is printed on the lower surface of the fourth layer dielectric plate corresponding to the lower surface of the third layer and is also positioned on both sides of the feeding hole and the short-circuit hole, while, at the same time, the metal patch leaves gaps at either side of the feeding hole and the short-circuit hole. Adding the metal patch twice allows one to increase capacitance coupling in order to change the dielectric constant of the dielectric plate. At the same time, it also plays a role in adjusting the matching impedance, as shown in Figure 3d. The fifth layer dielectric plate mainly prints a metal copper-clad connection ground plate on the lower surface of the fifth layer dielectric plate to remove the common mode resonant surface wave for the whole short circuit of the antenna. The second through to the fifth layers were adhered together using FR4. The second to fifth dielectric plates are mainly used for supporting feeding lines and short-circuit holes formed by through holes. When the antenna array performs beam scanning, refraction will occur on the dielectric surface, causing surface wave interference and resulting in the increase of scanning blind area and internal impedance. Therefore, in the antenna design, holes are used in the second to fifth dielectric plates to reduce the surface wave interference and adjust the impedance. The low-frequency bandwidth is widened by adding short-circuit holes, rectangular metal sheets, dielectric plate drilling holes, etc., which makes the low-frequency absorbing ability stronger and ensures the width of the high-frequency bandwidth. Through the above structure, we use CST Microwave Studio 2023 to perform full-wave simulation and optimization of the antenna unit structure. The optimized dimensional parameters of the TCDA unit structure are shown in Table 1. The amplitude of *S*_11_ (Figure 4) obtained by the full-wave simulation is −10 dB in the frequency band of 4.14 GHz–13 GHz, which shows that the designed antenna unit has good impedance matching at ultra-wide frequency operating ranges.

### 2.3. Design and Simulation of MRFES Unit Connected with Absorption Circuit

**Figure 5:**
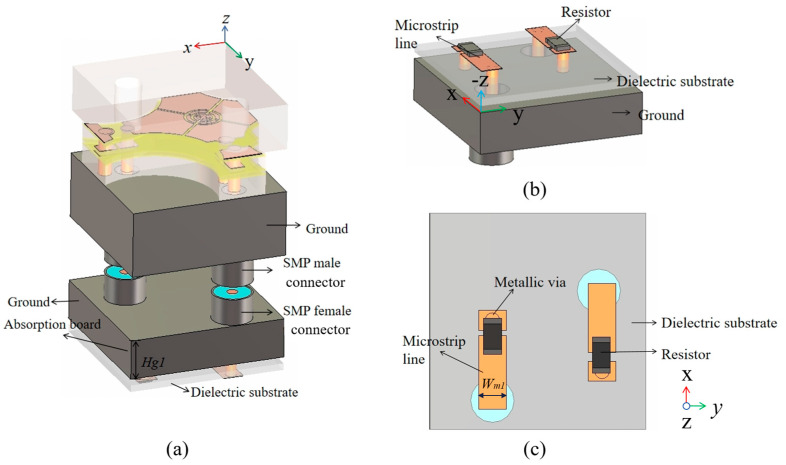
(**a**) The 3D diagram of the proposed absorptive EM surface structure, (**b**) the absorptive circuit board, and (**c**) top view of the absorptive circuit board.

**Figure 6:**
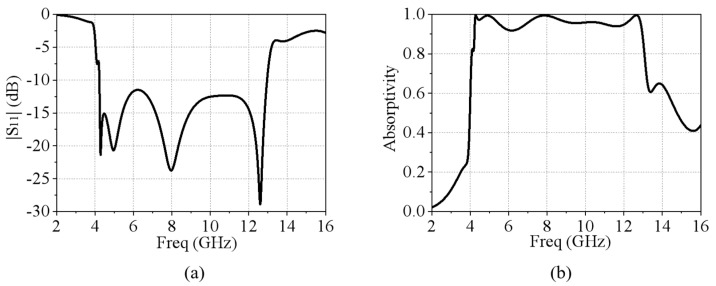
(**a**) The simulated magnitude of *S*_11_ of the absorptive EM surface and (**b**) the simulated absorptivity.

As mentioned above, when the TCDA is back-connected with an absorbing circuit board, the entire absorptive EM surface is formed. The complete absorbing EM surface unit structure is shown in Figure 5a. The basic structure involves the TCDA and the back-connected RF circuit. In the RF circuit design, the absorbing circuit is mainly composed of a metal microstrip line and a lumped resistor. The terminal of the microstrip line is connected with the lumped resistor to absorb the introduced EM wave signal as shown in Figure 5b,c. The dielectric layer of the absorbing circuit is Rogers5880 and the dielectric constant is 2.2. For impedance matching, the width of the microstrip line is set to *W_m_*_1_ = 1.52 mm and the resistance of the lumped resistor is 50 Ω. The TCDA on the upper surface is used to receive and transmit electromagnetic waves, and the back-loaded RF circuits are composed of resistors that absorb received electromagnetic waves. When the EM wave is incident on the unit surface, the antenna on the top couples the incident EM wave into the absorbing circuit within the working bandwidth. The received EM wave signal is then absorbed by the resistor at the terminal of the circuit. In CST Microwave Studio 2023, the above structure is simulated with periodic boundary conditions in the x and y directions and open add space boundary conditions in the z direction. The simulated results of the magnitude of *S*_11_ are shown in Figure 6a. Note that the simulated results are exactly the same for both vertical and horizontal polarizations due to the symmetry of the unit structure. The absorptivity of this unit can then be calculated as follows:

*A* = 1 − |*S*_11_| (1)

**Figure 7:**
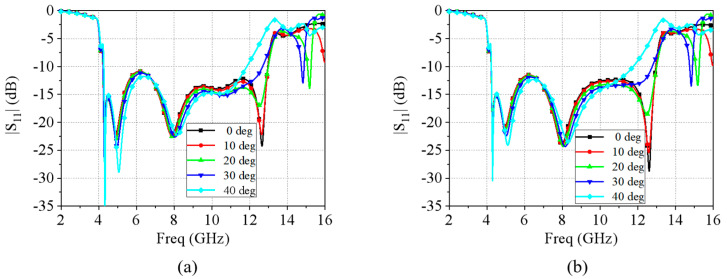
The wide-angle scanning simulation at 0°–40°. (**a**) TE polarization and (**b**) TM polarization.

It can be seen that, from 4.23 to 12.96 GHz, the absorptivity is greater than 90% at normal incidence. The absorption range reaches a bandwidth ratio of 3:1. This is a reduction of 1 GHz of operating bandwidth when compared with the operating range of the antenna unit. Additionally, because of the structure symmetry, the designed absorbing EM surface has dual polarization characteristics. The simulated results of *S*_11_ and absorptivity are exactly the same for both vertical and horizontal polarizations. At the same time, the wide-angle scanning of 0°–40° was also performed, as shown in Figure 7. Within the operating frequency band, the simulated results of *S*_11_ are below −10 dB at incident angles from 0° to 30°, but the bandwidth becomes significantly narrower at 40°.

### 2.4. Design and Simulation of MRFES Unit Connected with Polarization Conversion Circuit

**Figure 8:**
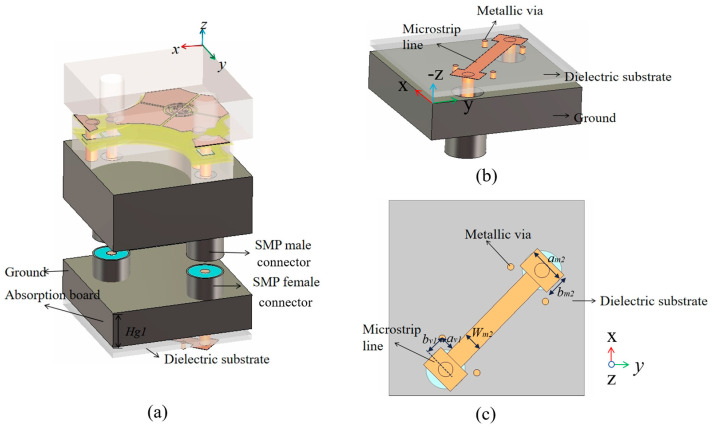
(**a**) The 3D diagram of the polarization conversion EM surface structure, (**b**) the 3D diagram of the polarization conversion circuit board and (**c**) the top view of the polarization conversion circuit board, where *a_v_*_1_ = 0.93 mm, *b_v_*_1_ = 1.2 mm, *W_m_*_2_ = 1.2 mm, *a_m_*_2_ = 2.3 mm, *b_m_*_2_ = 1.5 mm.

As previously mentioned, when the broadband antenna array is backed with a polarization conversion circuit board, it forms a polarization conversion EM surface. The overall unit structure is illustrated in Figure 8a. The polarization conversion circuit is primarily composed of metallic microstrip lines, with the circuit’s dielectric layer made of F4B and the dielectric constant of 2.2, as depicted in Figure 8b. One end of the polarization conversion circuit is connected with one of the feeding ports of the antenna through SMP connection, while the other end is connected to the left port. Each end of the circuit also has two metallic vias and a square patch for better vertical transition of coaxial line to microstrip line. By these cross-connected lines, the polarization conversion effect can be realized. The TCDA on the upper surface is used to receive and transmit electromagnetic waves, and the back-loaded RF circuits are used to transmit electromagnetic waves of one polarization to corresponding ports of another polarization. Assuming an *X*-pol EM wave impinges on this unit within the operating frequency band, the antenna structure first couples the incident EM wave into the coaxial line on the left. The polarization conversion circuit, based on the microstrip line layout, transmits the received EM signal to the coaxial line on the right, before feeding the tightly coupled antenna from this coaxial line. At this point, the tightly coupled antenna radiates the EM wave outward as a *Y*-pol wave, thus completing the reflective polarization conversion of the incident EM wave.

**Figure 9:**
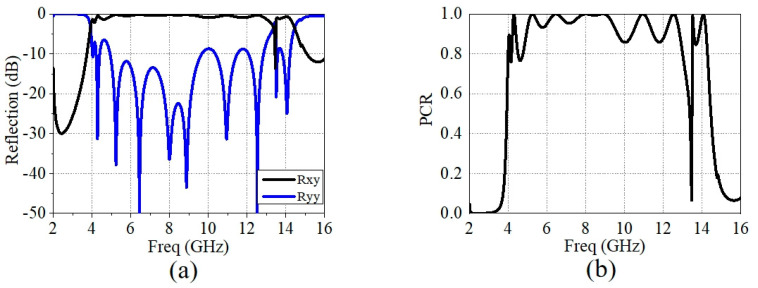
(**a**) The simulated results of the co-polarized and cross-polarized reflection of the polarization conversion EM surface. (**b**) The simulated results of PCR.

For the full-wave simulation of this polarization conversion EM unit, the same boundary conditions as those used for absorption/reflection were applied. Within the antenna’s operating bandwidth of 4.14 GHz to 13 GHz, the co-polarization reflection amplitude, as shown in Figure 9a, is predominantly below −10 dB from 4.93 GHz to 12.96 GHz, with slightly increased reflection amplitudes at two frequencies near 10 GHz and 11.78 GHz, indicating good reflection performance. The cross-polarization reflection amplitude, depicted in Figure 9a, demonstrates that, within the antenna’s operating bandwidth, from 4.84 GHz to 12.88 GHz, the reflection efficiency is above −1 dB for over 90% of the frequency range. Additionally, the bandwidth with cross-polarization efficiency higher than 90% covers 90% of the antenna’s operating bandwidth, according to the following formula for the polarization conversion ratio (PCR):

(2) $$PCR=\frac{|r_{xy}{|}^{2}}{\left(|r_{xy}{|}^{2}+|r_{yy}{|}^{2}\right)}$$

The simulated PCR results for the designed polarization conversion MRFES is shown in Figure 9b. It is evident that the PCR exceeds 0.9 in the frequency range of 4.85 GHz to 12.89 GHz, and that it remains above 0.83 from 4.17 GHz to 13 GHz, demonstrating its capability for broadband and efficient polarization conversion.

In summary, when the same broadband tightly coupled antenna array unit is back-loaded with a corresponding RF circuit board, it can achieve broadband absorption and polarization conversion effects independently. This theoretically validates the effectiveness of our proposed MRFES, which can realize reconfigurable EM wave manipulation with broadband working bandwidth. Compared with previous absorbing and polarization conversion metasurface designs [5,6,7,8,9], the MRFES structure employs a modularized design, meaning that it is convenient to replace the back-loaded RF circuit boards with different functions, thus realizing switchable broadband absorption and polarization conversion functions.

## 3. Physical Fabrication and Measurement

**Figure 10:**
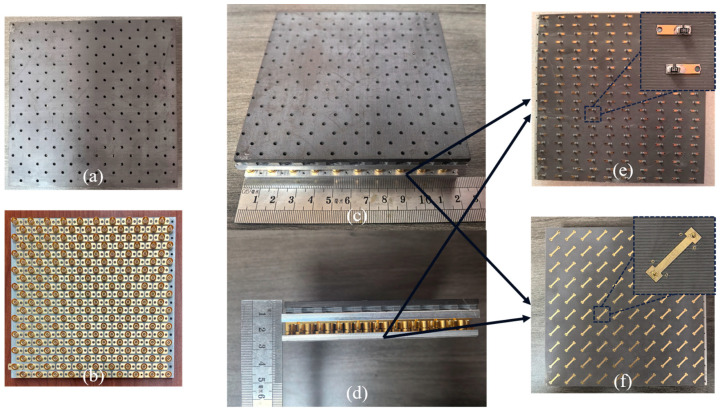
Photo of the fabricated prototype. (**a**) Front view of the TCDA array, (**b**) back view of the TCDA array, (**c**) full structure of the MRFES, (**d**) side view of the MRFES, (**e**) front view of the absorption circuit board, (**f**) front view of the polarization conversion board.

The proposed design for a reconfigurable EM surface capable of switching between broadband absorption and polarization conversion is realized by loading absorptive and polarization conversion circuit boards on to the back side of the broadband antenna array’s ground plane. In order to verify the effectiveness of the proposed scheme, we manufactured the physical prototype on the basis of the simulation model. In the antenna section, the dielectric substrate is made of F4B. The adhesive layer between substrates is FR4, with a thickness of 0.1mm and a dielectric constant of 4.4. The metal patch on the dielectric layer has a thickness of 0.035 mm. The fifth dielectric substrate layer, which requires grounding, has a full metal backing with isolated rings etched around every pin hole location. An aluminum plate serves as the ground plane, also functioning to support the dielectric substrates and secure the SMP connectors. The broadband antenna dielectric substrate, the absorptive circuit board, polarization conversion circuit board and the SMP connectors are fixed to their corresponding aluminum plates. The dielectric plate was fixed using an adhesive with a dielectric constant of 2.9. The SMP male connectors and the SMP female connectors are fastened to the aluminum plate through the hole flange. The SMP male connectors use a smooth-bore interface for convenient assembly and disassembly. During the assembly process, we use pressing and lifting mechanical tools for assistance to ensure that no damage is caused to the antenna and components. The physical photographs of the broadband antenna array and its components are depicted in Figure 10.

**Figure 11:**
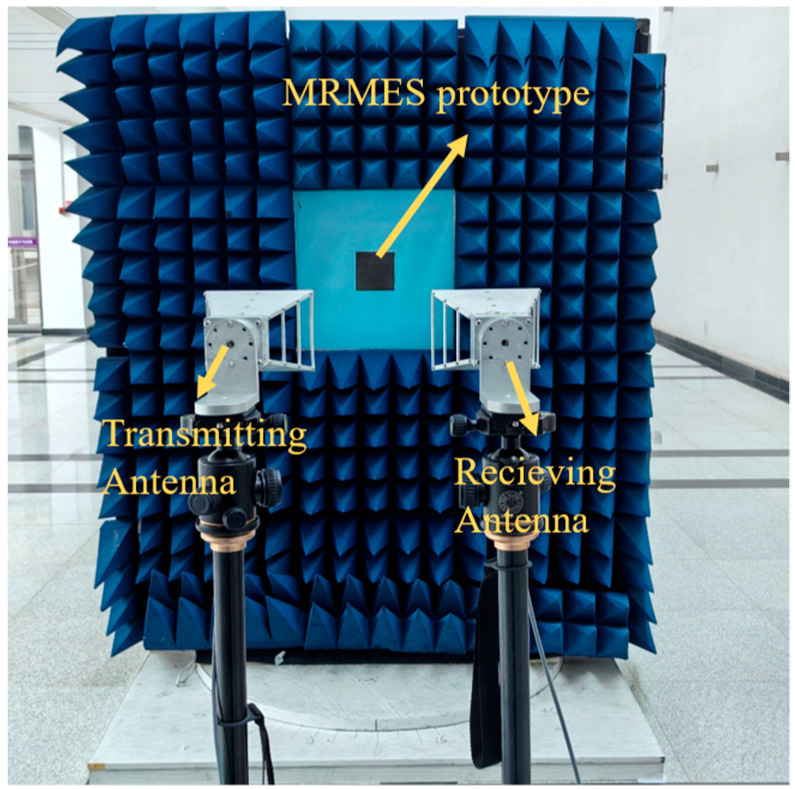
The measurement environment.

In practical testing, the prototype is centered in a measurement window surrounded by microwave absorbing material. The prototype support screen, except for the area where the sample is placed, is entirely covered with absorptive material to eliminate scattering and unwanted reflections. The measurement environment is illustrated in Figure 11. Two horn antennas, operating within the 2–18 GHz frequency band, are respectively configured as the transmitting and receiving antennas. These are fixed on two tripod stands and are connected to the two ports of a vector network analyzer (Agilent N9918A, which is manufactured by Keysight Technologies (formerly Agilent) in Santa Rosa, CA, USA) via low-loss coaxial cables. Both horn antennas and the sample center are aligned at the same horizontal level, situated on the same side of the sample support screen. The distance between the horn antennas and the sample complies with the far-field conditions stipulated by NRL standards.

In the testing procedure, a metal plate of the same size as the fabricated sample is initially positioned, and its S parameters are measured. Subsequently, the S parameters are measured with the sample plate in place. After importing the data into MATLAB R2018a for processing, the reflection coefficient of the sample is obtained.

**Figure 12:**
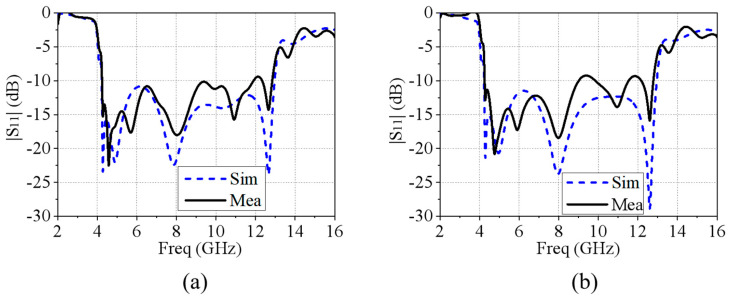
Comparison of the simulated and measured reflections of MRFES in absorbing state under (**a**) TE polarized (y-pol) incidence and (**b**) TM polarized (x-pol) incidence.

Figure 12 illustrates the simulated and measured results of the reflection amplitude for the EM surface connected with the absorptive circuit board under the normal incidence of (a) TE polarization (y-pol) and (b) TM polarization (x-pol). Both transmitting and receiving antennas are in the same polarization state. Within the 4 GHz to 13 GHz range, the reflection coefficient for the normal incident wave is less than −10 dB. Except for a few frequency points, the measured results are largely consistent with the simulated ones under both polarization conditions, confirming the dual-polarization characteristics and broadband absorptive features of the proposed MRFES structure in the absorbing state.

**Figure 13:**
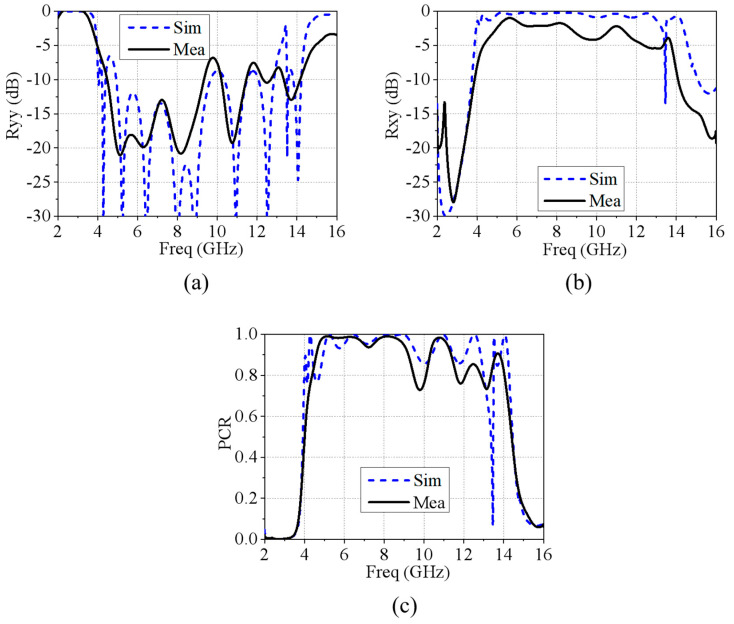
(**a**) Comparison of the measured and simulated results of the MRFES in polarization conversion state for co-polarized reflection, (**b**) the cross-polarized reflection, and (**c**) The PCR.

When testing the polarization conversion MRFES, it is necessary to measure the amplitude of the reflection coefficient for both co-polarization and cross-polarization. As shown in Figure 13, a comparison is displayed between the measured reflection coefficient amplitudes and the simulated results. We can conclude that, in co-polarization, the trends at high frequency points are similar, while at lower frequencies, though showing larger discrepancies, the trends still exhibit good performance with values below −10 dB. In cross-polarization, the discrepancy between the high and low frequency trends is greater, but the low frequency trend closely matches the simulation results. Additionally, and according to Equation (2), the PCR is calculated and is displayed in Figure 13c. From the comparison between the measured and the simulated results, it is evident that the trends in low frequency band are similar, the locations of high-frequency points are consistent and that the overall trends are alike.

In summary, we find that the overall trends between the actual measurements and full-wave simulation results are similar, albeit with some limited discrepancies. These discrepancies may arise from errors and tolerances in the practical environment of the experimental setup, the fabrication and the assembly of the physical prototypes. Besides the small size of the test samples (resulting in significant edge effects) and differences between the observed surfaces in experiments and simulations, the fact that the testing area is not entirely enclosed also decreases the accuracy of measurement. Considering all of these influences, the measured results are largely in line with the corresponding simulated ones. This validates the idea that the proposed design of the MRFES, when using TCDA and BLRFC, can indeed achieve switchable absorption and polarization conversion capabilities, while offering the benefits of ultra-wide bandwidth, high performance, and modularity.

## 4. Conclusions

This study introduces a modularized reconfigurable functional electromagnetic surface (MRFES) for broadband absorption and polarization conversion using tightly coupled dipole antennas (TCDA) and back-loaded radio frequency circuits (BLRFC). The MRFES structure employs a modularized design, consisting of a broadband antenna array panel and functional RF circuit boards. The antenna array initially receives the incident electromagnetic waves from space, coupling them into the feed line, which is then transmitted to the back-loaded RF circuit via coaxial cables. Due to the modularized design, it is convenient to replace the back-loaded RF circuit boards with different functions. When TCDA is connected with an absorptive circuit board, the entire MRFES can achieve broadband, dual-polarized absorption, with simulation results showing reflection amplitude below −10 dB in the 4 GHz to 13 GHz range. The measurement results are consistent with the simulation results. When connected with a polarization conversion circuit board, the MRFES can realize wideband polarization conversion. The simulation results demonstrate that the polarization conversion ratio (PCR) is greater than 0.9 in the 4.85 GHz to 12.89 GHz frequency band and greater than 0.83 in the 4.17 GHz to 13 GHz frequency band. The experimental results closely follow the simulation trends. These results validate the effectiveness of the proposed MRFES design methodology. This approach offers advantages such as flexible control, the independent design of the EM wave reception and control sections, broad bandwidth, and ease of integrating various active components and chips for the wave control. Our proposed design can achieve a wider bandwidth and better broadband ductility, and the cost is lower than that of chip integration. In the military field, this EM surface shows promising potential for application in stealth technology through its anti-interference ability and multi-functionality. In the civil field, the surface can improve the reliability and capacity of wireless communication. Thus, it has promise for a wide range of applications, including those of wireless communications, radar stealth, EM compatibility, and photonic devices.

## Figures and Tables

**Table 1:** Optimized structural parameters of the TCDA unit.

Parameter	*W*1	*W*2	*W*3	*L*1	*L*2	*L*3	*L*4	*H*2
Value/mm	2.5	5	3	1.1	3.25	2.3	3.17	0.035
Parameter	*R*1	*R*2	*R*3	*C*1	*C*2	*B*1	*H*1	*H*3
Value/mm	0.5	0.4	0.5	1.1	0.65	1.1	3	1.5

## Data Availability

The data presented in this study are available on request from the corresponding author.
